# MicroRNA Expression Profiling in Behçet's Disease

**DOI:** 10.1155/2018/2405150

**Published:** 2018-05-07

**Authors:** Antonio Puccetti, Andrea Pelosi, Piera Filomena Fiore, Giuseppe Patuzzo, Claudio Lunardi, Marzia Dolcino

**Affiliations:** ^1^Immunology Area, Pediatric Hospital Bambino Gesù, Viale San Paolo 15, 00146 Rome, Italy; ^2^Department of Experimental Medicine-Section of Histology, University of Genova, Via G.B. Marsano 10, 16132 Genova, Italy; ^3^Department of Medicine, University of Verona, Piazzale L.A. Scuro 10, 37134 Verona, Italy

## Abstract

**Background:**

Behçet's disease (BD) is a chronic inflammatory multisystem disease characterized by oral and genital ulcers, uveitis, and skin lesions. MicroRNAs (miRNAs) are key regulators of immune responses. Differential expression of miRNAs has been reported in several inflammatory autoimmune diseases; however, their role in BD is not fully elucidated. We aimed to identify miRNA expression signatures associated with BD and to investigate their potential implication in the disease pathogenesis.

**Methods:**

miRNA microarray analysis was performed in blood cells of BD patients and healthy controls. miRNA expression profiles were analyzed using Affymetrix arrays with a comprehensive coverage of miRNA sequences. Pathway analyses were performed, and the global miRNA profiling was combined with transcriptoma data in BD. Deregulation of selected miRNAs was validated by real-time PCR.

**Results:**

We identified specific miRNA signatures associated with BD patients with active disease. These miRNAs target pathways relevant in BD, such as TNF, IFN gamma, and VEGF-VEGFR signaling cascades. Network analysis revealed several miRNAs regulating highly connected genes within the BD transcriptoma.

**Conclusions:**

The combined analysis of deregulated miRNAs and BD transcriptome sheds light on some epigenetic aspects of BD identifying specific miRNAs, which may represent promising candidates as biomarkers and/or for the design of novel therapeutic strategies in BD.

## 1. Introduction

Behçet's disease (BD) is a rare and chronic multisystem disease characterized by a triple-symptom complex of recurrent oral aphthous ulcers, genital ulcers, and uveitis. Moreover, manifestations of vascular, articular, neurologic, urogenital, gastrointestinal, pulmonary, and cardiac involvement may occur. Hippocrates described BD in the fifth century BCE. In 1930, the Greek ophthalmologist Benediktos Adamantiades reported a patient with inflammatory arthritis, oral and genital ulcers, phlebitis, and iritis. The disease is named after the Turkish dermatologist Hulusi Behçet, who identified it in a patient in 1924 and published a description of the disease in 1937.

As virtually no unique histological or laboratory features have been identified to help in the diagnosis of the disease, clinical features are used to define and diagnose Behçet syndrome. An international study group on Behçet's disease has recently revised the criteria for the classification/diagnosis of BD [1].

There are sporadic cases of BD all around the world, but it is most frequently seen along the ancient Silk Route because of its frequency in the Middle East and far-east Asia (prevalence of 14–20/100,000 inhabitants), and these regions have traditionally been considered the endemic areas for the condition [2].

BD is a sporadic disease, but a familial aggregation is well known. Carriers of HLA-B51/HLA-B5 have an increased risk of developing Behçet's disease compared with noncarriers. HLA-B51 is the strongest associated genetic factor, and it has been shown to be more prevalent in Turkish, Middle Eastern, and Japanese populations, with a higher prevalence of Behçet's disease in these populations [3]. Non-HLA genes also contribute to the development of BD [3]. Genome-wide association studies have shown that polymorphisms in genes encoding for cytokines, activator factors, and chemokines are associated with increased BD susceptibility. Among cytokines, IL-10 polymorphisms cause a reduction in the serum level of IL-10, an inhibitory cytokine that regulates innate and adaptive immune responses; on the other hand, IL-23 receptor polymorphism, which reduces the response to IL-23 stimulation, is associated with protection from BD [3–5]. Recent data reported also associations with CCR1, STAT4, and KLRC4 encoding for a chemokine receptor, a transcription factor implicated in IL-12 and IL-23 signaling and a natural killer receptor [6, 7]. Finally, susceptibility genes involved in the innate immune response to microbial exposure have recently been identified by Immunochip analysis [8].

Increased Th1, CD4^+^ and CD8^+^ T cell, *γδ* ^+^ T cell, and neutrophil activities have been found both in the serum and in inflamed tissues of BD patients, suggesting the involvement of innate and adaptive immunity in the pathogenesis of BD [2, 9]. Studies on T lymphocytes have suggested a Th1-predominant response. Both CD4^+^ and CD8^+^ lymphocytes are higher in the peripheral blood, with increased levels of IL-2 and interferon- (INF-) *γ* cytokines [10]. The cytokine Th17 may also play an important role in the pathogenesis of the disease [2, 11]. This hypothesis is supported by the observation of high IL-21 and IL-17 levels in sera of patients affected by BD with neurologic involvement [12, 13]. Another study has reported a higher Th17/Th1 ratio in peripheral blood of patients with BD compared to healthy controls, and this ratio was higher in patients with uveitis or folliculitis compared with patients without these manifestations [14, 15].

MicroRNAs (miRNAs) are small noncoding RNAs that play an important role in the regulation of several biological processes through their interaction with cellular messenger RNAs [16]. Inflammatory responses have an impact on miRNA expression, regulating their biogenesis by altering the transcription and processing of precursor transcripts or influencing the stabilization of mature miRNAs [17, 18]. In recent years, the number of miRNAs implicated in immune system development and function has dramatically enhanced, and there has been widespread discussion of their potential use as therapeutics for immunological diseases [16]. Indeed, the aberrant expression of miRNAs frequently occurs in human diseases, including hematological disorders and autoimmunity [19, 20]. The concept that miRNAs participate in the pathogenesis of diseases, especially refractory diseases with unidentified mechanisms, might lead to a novel effective treatment. A number of studies have reported a differential expression of miRNAs in several inflammatory autoimmune diseases, such as in rheumatoid arthritis (RA), multiple sclerosis, systemic lupus erythematosus, psoriasis, and systemic sclerosis [21]. These studies highlighted a deep implication of miRNAs as regulatory molecules in autoimmunity and the intriguing possibility to use miRNAs as disease biomarkers in these immunological disorders.

As far as BD concerns, little is known about miRNA expression; moreover, no high-throughput miRNA expression studies have been conducted to identify miRNAs specifically associated with the disease and no study has been so far performed which combines the analysis of blood microRNAs with transcriptional profiles in patients with BD.

In the present study, we performed a miRNA microarray analysis on peripheral blood mononuclear cells (PBMCs) of BD patients. We identified specific miRNA signatures associated with patients with active BD. These deregulated miRNAs target signaling pathways typically implicated in BD pathogenesis, such as TNF, interferon gamma, and VEGF and VEGFR signaling cascades.

The modular analysis of differentially expressed genes in BD revealed pathogenetically relevant networks that are possibly targeted by the identified miRNAs. This study sheds light on some aspects of BD pathogenesis identifying deregulated miRNAs as promising candidates for the discovery of disease biomarkers and/or as molecular tools for designing novel therapeutic strategies in BD.

## 2. Materials and Methods

### 2.1. Patients

A group of 6 subjects with BD was used for the gene array study. All the patients attended the Unit of Autoimmune Diseases at the University Hospital in Verona, Italy.

All patients fulfilled the International Criteria for Behçet Disease (ICBD): oral aphthosis, genital ulcers, and ocular lesions were each given 2 points, whereas 1 point was assigned to each of skin lesions, vascular manifestations, and neurological manifestations. A patient scoring 4 points or above was classified as having BD [22, 23]. At enrollment, none of the patients had active infections or was affected by malignancies.

The clinical features of the patients are reported in Table 1 that also includes a description of the BD patients selected for the gene array study.

A written informed consent was obtained from all the participants to the study. The study was approved by the local Ethical Committee of the Azienda Ospedaliera Universitaria of Verona, Verona, Italy. All investigations have been conducted according to the principles expressed in the Helsinki declaration.

### 2.2. Microarray Analysis

Blood samples were collected in BD Vacutainer K_2_EDTA tubes using a 21-gauge needle. Peripheral blood mononuclear cells (PBMC) were obtained upon stratification on Lympholyte® cell separation density gradient (Cedarlane, Burlington, Canada). Total RNA extraction from PBMC was performed with miRNeasy Mini Kit following the manufacturer's protocol (Qiagen GmbH, Hilden, Germany). RNA concentration and purity were evaluated by spectrophotometric analysis (NanoDrop 2000; Thermo Fisher Scientific, Wilmington, DE, USA), and a further check for RNA integrity was performed with 2100 Bioanalyzer (Agilent Genomics, Santa Clara, CA, USA) before microarray hybridization. Sample hybridization and scanning were performed as recommended by the Affymetrix (Affymetrix; Thermo Fisher Scientific Inc.) supplied protocols, by the Cogentech Affymetrix Microarray Unit (Campus IFOM-IEO, Milan, Italy), and Affymetrix GeneChip® miRNA 4.0 (Affymetrix; Thermo Fisher Scientific Inc., Waltham, MA, USA) was used. The GeneChip miRNA 4.0 arrays contain more than 30,000 probes including those encoding for 2578 mature human miRNAs, according to Sanger miRBase v.20.

The arrays were analyzed employing the Transcriptome Analysis Console (TAC) 4.0 software (Applied Biosystems, Foster City, CA USA, by Thermo Fisher Scientific, Waltham, MA, USA). The Signal Space Transformation- (SST-) Robust Multiarray Average (RMA) algorithm was applied to background-adjust, normalize, and log-transform signal intensity.

Relative expression levels of each microRNA were validated applying a one-way analysis of variance (ANOVA) and false discovery rate (FDR) correction (*p* ≤ 0.01). MicroRNAs with an expression level of at least 1.5-fold different in the test sample versus control sample were analyzed.

Targeted genes of significantly modulated miRNAs were identified using the integrative database for human microRNA target predictions mirDIP [24]. All the source filters and a very high confidence class were applied for our analyses.

Pathway enrichment analysis of miRNA gene targets and differentially expressed genes (DEGs) in Behçet's disease was carried out using FunRich (http://www.funrich.org/) [25], and only Bonferroni-corrected enriched *p* values ≤0.01 calculated by the hypergeometric test were considered.

Pathways enrichment analysis of DEGs in BD was also performed employing the Panther expression analysis tools (http://pantherdb.org/) [26].

### 2.3. Protein-Protein Interaction (PPI) Network Construction and Network Clustering

Differentially expressed genes (DEGs) in BD samples from our previous analyses (Puccetti et al. unpublished observations) were mapped to the STRING database (version 1.0; http://string-db.org/) [27] to detect protein-protein interactions (PPI) pairs validated by experimental studies. A score of ≥0.7 for each PPI pair was selected to construct the PPI network. For the topological analysis of the built network, Cytoscape software was used and network clustering analysis was performed with the MCODE plugin of Cytoscape, based on the thresholds of module score > 1.5 [28].

### 2.4. Real-Time PCR

Mature miRNA expression was assayed by TaqMan® Advanced miRNA assay chemistry (Applied Biosystems, Foster City, CA, USA). Briefly, 10 ng of total RNA was reverse transcribed and preamplified with TaqMan Advanced miRNA cDNA synthesis kit following the manufacturer's instructions (Applied Biosystems, Foster City, CA, USA). Preamplified cDNA was diluted 1/10 in nuclease-free water, and 5 *μ*L of diluted cDNA for each replicate was loaded in PCR. 20 *μ*L PCR reactions were composed by 2x Fast Advanced Master Mix and TaqMan Advanced miRNA assays for hsa-miR-143-3p (477912_mir), hsa-miR-199a-5p (478231_mir), and hsa-miR-4505 (477842_mir). The mean of Ct for hsa-miR-16-5p (477860_mir) and hsa-miR-26a-5p (477995_mir) expression was used to normalize miRNA expression. Real-time PCR was carried out in triplicate on a QuantStudio 6 Flex instrument (Applied Biosystems, Foster City, CA, USA). Expression values were reported as fold change with respect to healthy controls by the ΔΔCt method using QuantStudio Real-Time PCR system software versus 1.3.

## 3. Results

### 3.1. High-Throughput MicroRNA Expression Profiling in Peripheral Blood Mononuclear Cells of Behçet's Disease

Since a global miRNA expression analysis in BD with an updated coverage of miRNA sequences has not been performed yet, we wanted to provide a careful description of miRNAs associated with BD by interrogating the transcription of a large amount of different microRNA sequences in BD PBMCs by microarray strategy.

Therefore, PBMCs derived from 6 patients with BD and 6 healthy age- and sex-matched donors were analyzed using a dedicated and high-density array with a coverage for more than 2500 human microRNA transcripts and all mature miRNA sequences in miRBase Release 20. The clinical features of patients included in the microarray study are reported in Table 1.

Microarray analysis revealed a high number (269) of modulated miRNAs that satisfied the FDR-corrected *p* value criterion (*p* ≤ 0.01) and the fold change criterion (FC ≥ ∣1.5∣), showing a robust and statistically significant variation between BD and healthy control samples (Supplementary Table 1). Such a large number of modulated transcripts clearly reflect the high performance of the array in the detection of a wide range of microRNA sequences. We thereafter sharpened our analysis by selecting only modulated miRNAs annotated as “high confidence” in miRBase 21 (http://www.mirbase.org), and to make our results more informative, we further narrowed down the analysis to miRNAs for which gene targets were annotated in FunRich.

By these criteria, we selected 47 modulated miRNAs that are shown in Table 2. Interestingly, almost all (45/47) miRNAs were down-modulated with only two up-regulated microRNAs.

**Figure 1:**
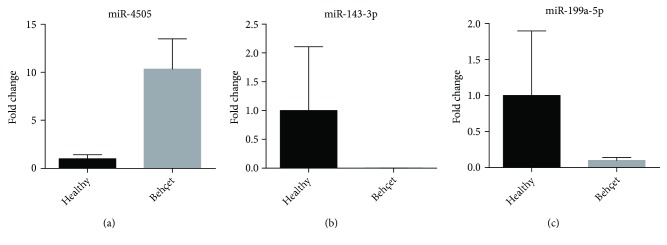
Validation of differentially expressed miRNAs by real-time PCR. Real-time PCR for the indicated miRNAs were performed in healthy controls (Healthy) and in BD patients (Behçet). Values are calculated as fold change with respect to healthy samples with the ΔΔCt method. miR-16-5p and miR-26-5p were used as endogenous controls for miRNA expression (see Material and Methods). Histograms indicate mean values; bars indicate standard deviation (SD). *p* = 0.05 (Mann–Whitney test).

Selected miRNAs significantly deregulated in the microarray analysis were validated by real-time PCR in the entire series of patients analyzed (see Figure 1).

### 3.2. Pathway Enrichment Analysis of miRNAs Deregulated in Behçet's Disease

In a second part of our analysis, we wanted to identify all the molecular pathways that were targeted by the selected miRNA performing a pathway enrichment analysis based on annotated gene targets in FunRich. The software allows to evaluate the miRNA regulatory effect and to identify controlled pathways based on predicted and/or validated miRNA-target interactions. We also applied a pathway enrichment analysis on the dataset of differentially expressed genes (DEGs) obtained in our previous study of gene expression profiling in BD (Puccetti et al., unpublished observations). In this study, we were able to select modulated genes that may play an important role in BD pathogenesis since they are involved in biological processes strongly connected to the typical features of the disease.

**Figure 2:**
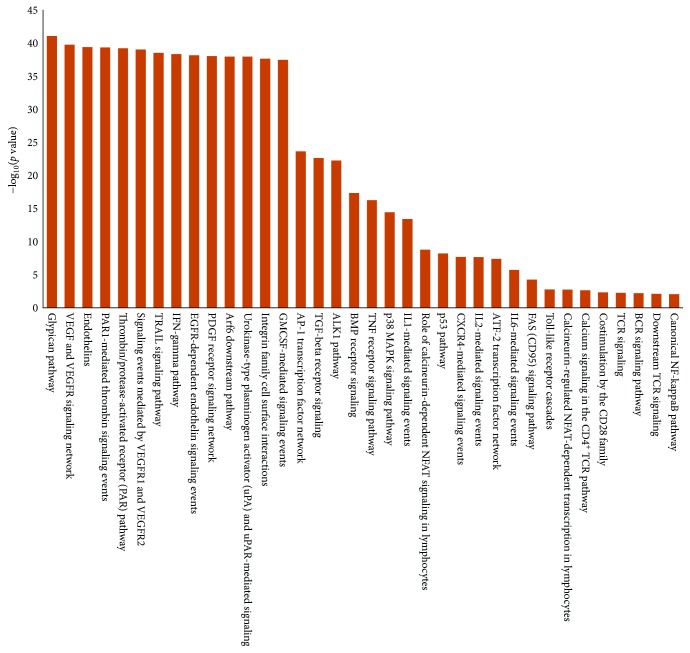
Histogram representing pathways enriched in BD-modulated miRNA targets genes and in BD DEGs. *y* axis: −log_10_(*p* value) (hypergeometric test).

Despite the strong statistical stringency applied to the two datasets (*p* ≤ 0.01), we obtained a high number of significantly enriched pathways both from the miRNA target datasets (277) and from the BD DEGs (164) (Supplementary Tables 2 and 3). Notably, we found that a large proportion of pathways from the BD DEGs was also enriched in the list of miRNA-validated targets (64%, 106/164; Supplementary Table 4), indicating that the selected miRNAs exert a strong impact on the molecular pathways altered in the disease. Moreover, the large number of enriched pathways clearly reflected the multisystemic involvement typical of Behçet's disease.  Figure 2 shows a graphical representation of selected commonly enriched pathways. Interestingly, the enriched categories were involved in vascular biology (i.e., glypican pathway, vascular endothelial growth factor, VEGF and VEGFR network, endothelin pathway, PAR1-mediated thrombin signaling events, thrombin/protease-activated receptor (PAR) pathway, EGFR-dependent endothelin signaling events, platelet-derived growth factor (PDGF) receptor signaling network, and urokinase-type plasminogen activator (uPA), and uPAR-mediated signaling) and in apoptosis (TRAIL, p53, and FAS signaling pathways). In addition, other relevant pathways enriched in the two datasets were related to the immune response (i.e., IL6-mediated signaling events, TCR and BCR signaling, calcineurin-regulated NFAT-dependent transcription in lymphocytes, and toll-like receptor cascades) and to the inflammatory response (i.e., tumour necrosis factor, TNF receptor, IFN-gamma, p38 MAP kinase, pathway, and IL1- and CXCR4-mediated signaling events.

### 3.3. Comparative Analysis of Selected miRNA Gene Targets and Differentially Expressed Genes in BD

**Figure 3:**
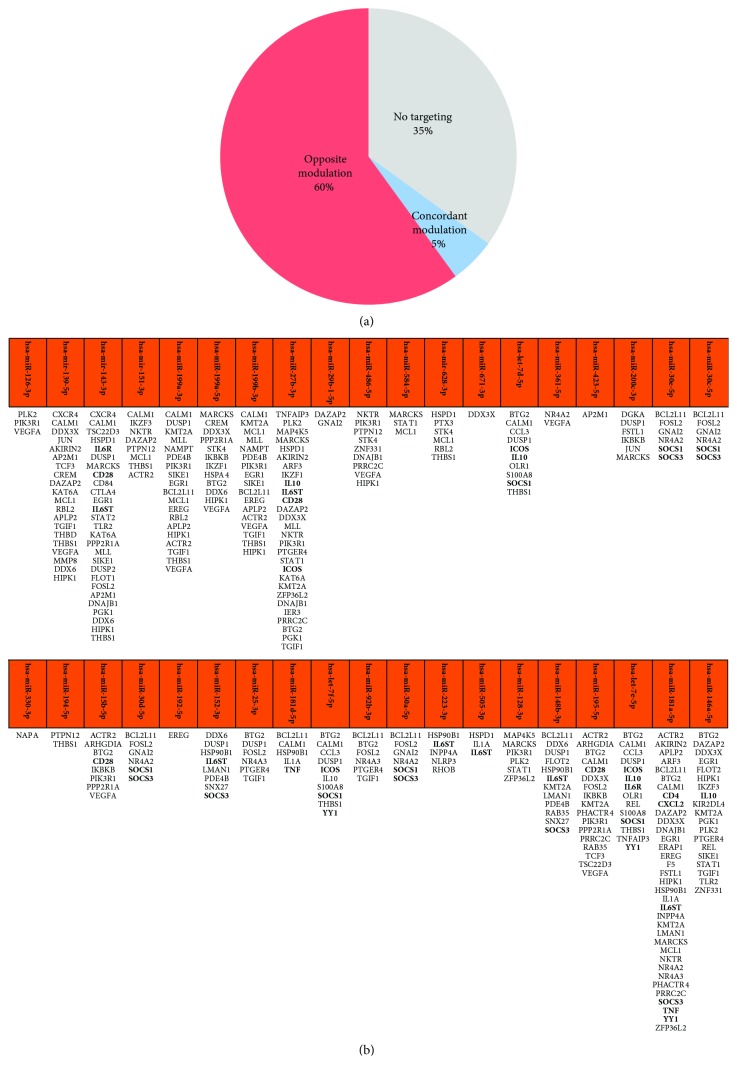
Selection of miRNAs that targeted genes differentially expressed in BD. (a) Pie chart showing the percentage of BD DEGs that were targeted by miRNAs modulated in BD. (b) BD-modulated miRNAs and their respective targeted BD DEGs. Genes associated with Th17 cells are written in bold characters.

To better define the role played by miRNAs in BD pathogenesis, we wanted to select miRNAs that were able to target genes modulated in BD. Therefore, we used a more sophisticated integrative database for human microRNA target predictions (mirDIP) [24] to obtain the lists of genes that were targeted, with a very high score class, by each of the selected miRNAs. Then, we compared the resulting target lists to genes differentially expressed in BD from our previous study (Puccetti et al., unpublished observations) and we observed that 65% of DEGs were targeted by the selected miRNAs. Interestingly, the vast majority of these DEGs showed an opposite modulation with respect to the relative targeting miRNA (Figure 3(a)), consistently with the typical role of miRNAs as negative regulator of gene expression (i.e., typically, up-regulated genes are targeted by down-modulated miRNAs and underexpressed genes are targeted by overexpressed miRNAs). All the above-mentioned miRNAs are presented in Figure 3(b) along with the compiled lists of their targets DEGs.

**Figure 4:**
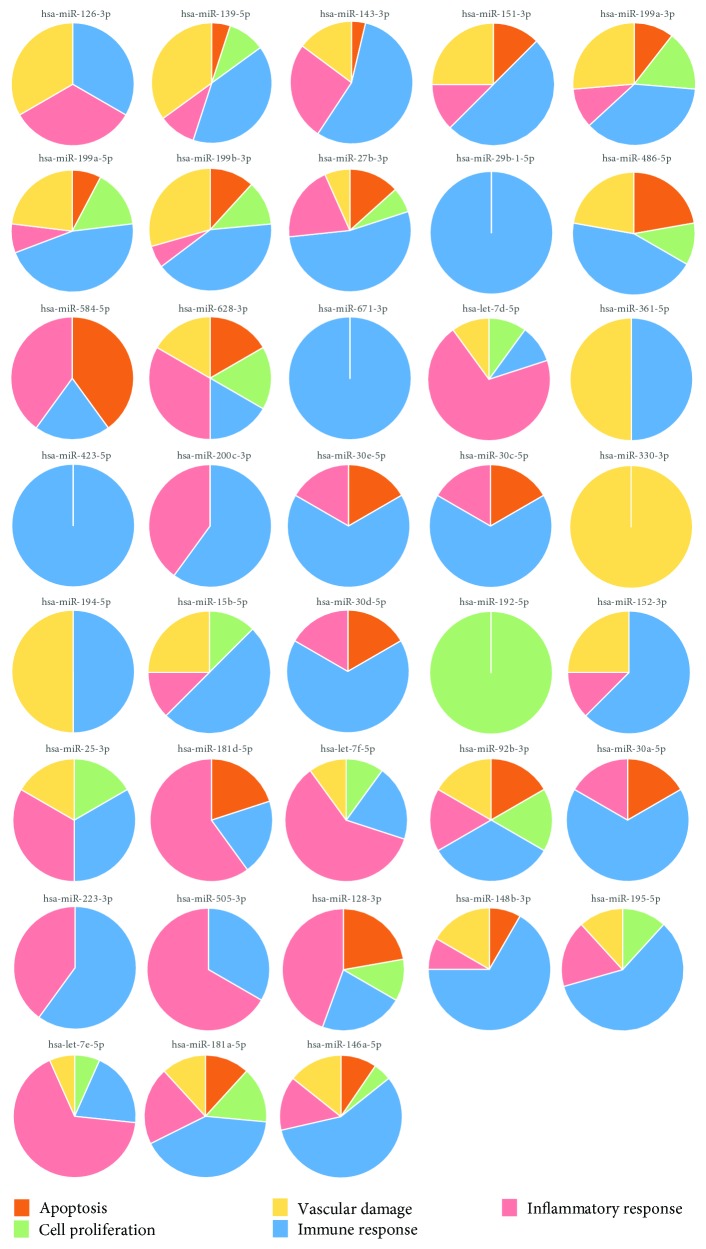
Functional classification of BD DEGs targeted by selected miRNAs. Pie charts showing the different GO biological processes in which BD DEGs targeted by selected deregulated miRNAs in BD can be classified.

All these selected miRNAs targeted genes involved in biological processes implicated in the disease pathogenesis including apoptosis, immune response, inflammation, and vascular damage (Figure 4). Thus, we could identify microRNAs that may control the gene modulation involved in the disease pathogenesis.  Table 3 shows all the targeted genes and their corresponding targeting miRNAs.

Selected miRNAs targeted many DEGs that sustained the inflammatory response typically associated with BD including TNF, IL1A, IL10, IL6R, CXCL2, CXCR4, TNFAIP3, OLR1, S100A8, HSP90B1, and CCL3 (see Figure 3(b) and Table 3). Interestingly, TNF (targeted by hsa-miR-181d-5p and -181a-5p), CCL3 (targeted by hsa-let-7e-5p, -7d-5p, and -7f-5p), IL10 (targeted by hsa-let-7f-5p, -7e-5p, -7d-5p, -miR-146a-5p, and -27b-3p), and IL1A (targeted by hsa-miR-505-3p, -181d-5p, and -181a-5p) have been detected at increased concentrations in sera or plasma of BD patients when compared to normal subjects [29–31]. Moreover, down-modulated miRNAs targeted DEGs involved in the adaptive immune response including genes that played a role in T and in B cell immune response. Among these genes, several miRNAs targeted CD28 (hsa-miR-143-3p, -27b-3p, -15b-5p, and -195-5p), CD4 (hsa-miR-181a-5p), ICOS (hsa-miR 27b-3p and -let-7f-5p, -7e-5p, and -7d-5p), CTLA4 (hsa-miR-143-3p), EGR1 (hsa-miR-143-3p, -146a-5p, -181a-5p, -199b-3p, and -199a-3p), and AKIRIN2 (hsa-miR-139-5p, -27b-3p, and -181a-5p). Interestingly, 18 miRNAs targeted genes of the TH-17 gene signature including CD28, CD4, ICOS, SOCS3, IL6ST, YY1, IL6R, TNF, CXCL2, and SOCS1 (Figure 3(b)).

In addition, selected miRNAs could also control DEGs involved in the innate immune response including, for example, NKTR (hsa-miR-181a-5p, -486-5p, -151-3p, and -27b-3p), KIR2DL4 (hsa-miR-146a-5p), STAT1 (hsa-miR-584-5p, -27b-3p, -128-3p, and -146a-5p), STAT2 (hsa-miR-143-3p), and DEGs belonging to the Toll-like receptor (TLR) pathway such as TLR2, IKBKB, JUN, REL, NAMPT, HSP90B1, HSPA4, and ARF3. In particular, we have to mention that TLR2 (targeted by hsa-miR-143-3p and -146a-5p) is thought to be up-regulated in BD patients [32, 33]. As many as 22 miRNAs targeted genes of the type I interferon signature like VEGFA, DDX3X, and the above-mentioned STAT1, STAT2, SOCS1, and IL10. The coexpression of miRNAs targeting DEGs involved in the type I IFN signaling and Th-17 related genes may reflect the presence of a synergy between IFN and Th17 pathways that is typical of autoimmune diseases [34–43], thus suggesting that an autoimmune mechanism can be involved in the BD pathogenesis.

Noteworthily, several miRNAs targeted all the eight genes involved in the JAK/STAT signaling pathway that we found up-regulated in our previous work (Puccetti et al., unpublished observations) including PIK3R1 and the above-mentioned STAT1, STAT2, IL6ST, IL6R, IL10, SOCS1, and SOCS3. The downmodulation of miRNAs targeting this pathway further supports the hypothesis of an autoimmune origin of BD since its activation is very frequently associated with autoimmune diseases [44]. Moreover, the JAK/STAT signaling pathway is active in CD4^+^ T cells of patients with BD [45].

Interestingly, modulated miRNAs targeted DEGs involved in the vascular damage associated with BD that is characterized by myointimal proliferation, fibrosis, and thrombus formation [46]. Indeed, several miRNAs targeted DEGs associated with blood coagulation (i.e., THBS1, F5, and LMAN1; see Table 3), a process whose alteration is typically associated with BD vasculitis. Noteworthily, 10 down-modulated miRNAs (hsa-let-7d/7e/7f-5p, hsa-miR-151-3p, -628-3p, -139-5p, 143-3p, -194-5p, -199a-3p, and -199b-3p) targeted THBS1, suggesting a crucial role of THBS1 in the pathogenesis of vascular damage. Other modulated miRNAs targeted DEGs that played a role in angiogenesis including MMP8, VEGFA, NR4A3, and NAPA. Moreover, NR4A3 is a transcription factor involved in vascular development [47] and NAPA promotes vascular endothelial- (VE-) cadherin localization at endothelial junctions [48]. Interestingly, increased serum levels of soluble VE-cadherins have been detected in BD patients [49]. Among the above-mentioned genes, we have to notice that VEGFA was predictively targeted by a high number (9) of down-modulated miRNAs, including hsa-miR-199a-3p, -15b-5p, -195-5p, -361-5p, -126-3p, -486-5p, -199a-5p, 199b-3p, and hsa-miR-139-5p. Other DEGs involved in vascular damage and targeted by modulated miRNAs were FOSL2, THBD, and PTX3. THBD, targeted by hsa-miR-139-5p, is increased in sera of BD patients compared with healthy subjects, and PTX3, targeted by hsa-miR-628-3p, is considered as a marker of small vessel vasculitis [50].

Moreover, DEGs involved in the apoptotic process were targeted, and among these, we can cite MCL1, DNAJB1, BCL2L11, ARHGDIA, ZNF331, and IER3 (Table 3). Finally, several modulated miRNAs targeted DEGs that played a role in cell proliferation, a process that can be induced in response to both apoptosis and to skin ulcer formation. Among these miRNAs, we can mention those that targeted BTG2, EREG, PRRC2, and APLP2. In particular, EREG (targeted by hsa-miR-199a-3p, -199b-3p, and -181a-5p and by hsa-miR-192-5p) and APLP2 (targeted by hsa-miR-199a-3p, -199b-3p, -139-5p, and -181a-5p) were involved in the proliferation of corneal epithelial cells and in corneal epithelial wound healing, respectively [51, 52]. Thus, this gene regulation may have a role in ocular manifestations of BD like keratitis.

### 3.4. Network Analysis of Genes Differentially Expressed in BD

**Figure 5:**
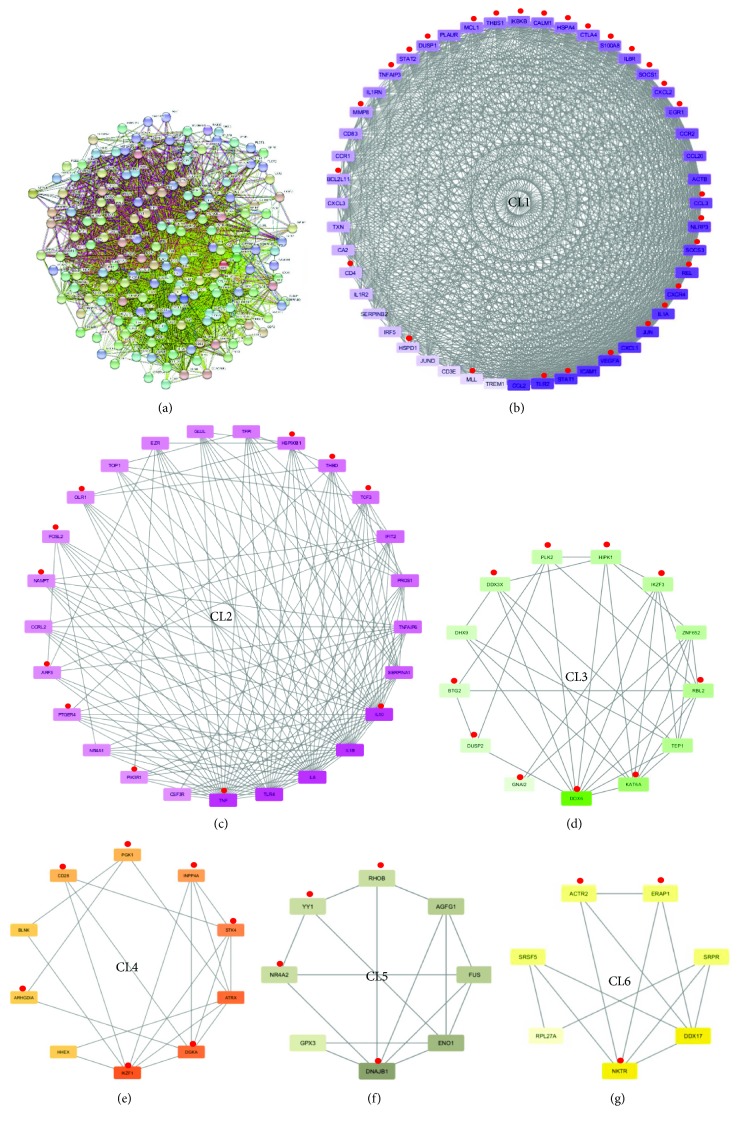
Network analysis of genes modulated in Behçet's disease. (a) PPI network of differentially expressed genes in BD. (b–g) Graphical representation of the six clusters that were extracted from the PPI network of modulated genes in BD. Red dots indicate DEGs that are targeted by modulated miRNAs in BD.

We performed a network analysis in which the functional interactions between the protein products of modulated genes in BD were evaluated. By this approach, a protein-protein interaction (PPI) network comprising 171 genes (nodes) and 3272 pairs of interactions (edges) was constructed (Figure 5(a)). We then performed a clustering analysis to identify areas of densely interconnected nodes (clusters/modules; CL) that are predicted to be involved in common biological processes and to have a crucial role in the disease pathogenesis. We could detect six clusters that are graphically represented in Figures 5(b)–5(g). Comparing the list of miRNA targets to DEGs included in the six clusters (Supplementary Table 5), we found that, in each cluster, a large number of DEGs were targeted (Supplementary Table 6 and Figures 5(b)–5(g)). In particular, we observed that several of these genes were involved in immune and inflammatory responses including TNF (CL2), IL10 (CL2), and IL1A (CL1). Moreover, many of such genes played a role in B cell response, like EGR1 (CL1), or in T cell response like, for example, CTLA4, CD4, and MLL (CL1); DUSP2 (CL3); and CD28 (CL4) and NR4A2 (CL5). Other DEGs targeted in clusters were involved in TLR signaling including HSPA4, IKBKB, JUN, REL, S100A8, TLR2 (CL1), and ARF3 and NAMPT (CL2). Interestingly, in the six clusters, we also observed that various genes involved in angiogenesis and/or in vascular damage were targeted by selected miRNAs including THBS1, MMP8, VEGFA (CL1), FOSL2, THBD (CL2), HIPK1 and DDX6 (CL3), PGK1 (CL4), and ACTR2 (CL6).

Given the well-known biological significance of highly connected gene clusters, to gain insight on the most relevant signaling networks that were controlled by the deregulated miRNAs, we performed a pathway enrichment analysis on targeted genes that were present in the six clusters. All the above-mentioned enriched pathways are listed in the Supplementary Table 7, and Table 4 shows a selection of the most relevant enriched signaling networks.

The TLR and the JAK/STAT signaling networks, two pathways notoriously involved in immune response and frequently associated with autoimmunity, were enriched in CL1-targeted genes (CL1-TGs). Other pathways implicated in the immune response were enriched in several genes targeted in clusters including T cell activation (CL1-TGs, CL2-TGs, and CL4-TGs), B cell activation (CL1-TGs), and PI3K signaling (CL2-TGs and CL3-TGs). This pathway is a crucial element in the regulation of adaptive and innate immune response [53, 54] and is a key player in inflammatory response. Therefore, in recent years, highly selective inhibitors of PI3K have been developed for anti-inflammatory treatments [55]. An enrichment in signalings involved in the inflammatory response was also found in CL1-TGs (inflammation mediated by chemokine and cytokine and interleukin pathways) and in CL5-TGs (integrin pathway).

In CL1-TGs and in CL3-TGs, the oxidative stress response pathway was enriched. Interestingly, an increased oxidative stress has been described in BD and it has been correlated to the severe inflammatory and degenerative clinical manifestations of the disease [56]. Several networks clearly connected to vascular damage were enriched in cluster TGs including PDGF, angiogenesis (CL1-TGs and CL5-TGs), Wnt, blood coagulation, endothelin, VEGF (CL2-TGs), and cadherin (CL6-TGs) signaling pathway. VE-cadherins have a crucial role in endothelial barrier integrity, and noteworthily, in CL6 the ACTR2 gene was targeted, known to stabilize adherens junctions between endothelial cells of the vascular wall interacting with cadherins [57]. Moreover, the Wnt signaling pathway is critically involved in vascular biology [58]. Finally, in CL1-TGs and in CL2-TGs the apoptosis and the p53 pathways were enriched, respectively.

## 4. Discussion

A systematic analysis of miRNA expression profiles in BD has not been performed yet. The aim of our work is therefore to provide a compiled description of miRNAs associated with BD using an array able to query a very large number of transcripts in the attempt to dissect the possible regulatory effects exerted by these molecules on the molecular pathways relevant for the disease pathogenesis.

In a previous work, we investigated BD-associated transcriptional profiles by a gene expression analysis of PBMC derived from BD patients and identified a gene modulation strictly connected to the disease pathogenesis (Puccetti et al., unpublished observations). Moreover, we showed the presence of a type I interferon and Th-17 gene signature, which suggests an autoimmune origin of BD. In this study, we aimed to complement this gene expression analysis detecting modulated miRNAs that may target differentially expressed genes (DEGs) identified in our previous analysis.

To this purpose, we used a sophisticated target prediction system (mirDIP) [24] to obtain the list of miRNA targets, which was then compared to the list of DEGs identified in blood samples of BD patients. The comparison of the two analyses (gene expression and miRNAs) allows selecting within deregulated miRNAs only those related to the modulation of genes that would be effectively altered in the PBMCs of BD patients and might have therefore a pivotal role in the pathogenesis of the disease.

We observed that there was a good overlap (60%) between the selected miRNA targets and the BD-modulated genes, indicating that the majority of the identified miRNAs regulate genes differentially expressed in BD. Such target genes belonged to functional classes strictly connected to typical features of BD. Indeed, several miRNAs targeted proinflammatory genes such as TNF and IL1A. Moreover, transcripts involved in both adaptive and innate immune response were also targeted. In this regard, we have to mention that selected down-modulated miRNAs controlled several Th17 cell-associated genes and transcripts involved in type I interferon response that we found up-regulated in our previous analysis. This may suggest a loss of control in BD on two synergistic mechanisms typically associated with an autoimmune response. We moreover observed a downregulation of miRNAs that control members of TLRs and JAK/STAT pathways, two molecular signalings involved in autoimmune diseases [44, 59] that are also active in BD [32, 60, 61]. In addition, miRNAs target genes involved in angiogenesis, and in blood coagulation, two processes commonly associated with BD vasculitis were also down-modulated in BD samples.

Consistently with the tight correspondence between deregulated miRNA and DEGS in BD, we observed a good overlap (64%) between the pathways enriched in the deregulated miRNA targets and in the genes differently expressed in BD. This indicated that, globally, the pathways identified by the selected miRNAs reflected fairly well the gene regulation described in our gene expression study, suggesting that the criteria applied for the selection of miRNAs had effectively identified miRNAs able to explain the gene modulation which we had previously described.

Interestingly, we found that both in BD-miRNA and in BD-DEGs dataset, meaningful signaling networks including, for example, VEGF and VEGFR, endothelins, PDGF receptor, TNF receptor, and IL1- and IL6-mediated TCR and BCR pathways were enriched.

A pivotal task in molecular biology is to understand gene modulation in the context of biological networks. Indeed, proteins achieve their functions in the protein-protein-interacting network, and interestingly, the dynamics of such networks can be influenced by miRNAs [62]. We therefore wanted to identify significant relationships among modulated miRNAs and the PPI network in which the protein product of modulated genes in BD can be involved. Moreover, since it is known that the repressive effect of a miRNA may lead to more severe biological effects when it is exerted on proteins with more interacting partners [62], to highlight more efficient interrelations, we focused our attention on the most connected proteins of the network, inspecting the presence of miRNA targets inside the six clusters extracted from the PPI network. We found that the six clusters identified were extensively targeted by several modulated miRNAs, thus indicating that the deregulation of selected miRNAs may have a meaningful effect on the dynamics of a protein network that control the disease pathogenesis. Indeed, genes targeted in the six clusters played an important role in vascular biology (i.e., VEGFA, THBS, THBS1, etc.), in inflammation (i.e., TNF, IL1A, IL6R, CXCR4, etc.), and in immune response (i.e., CD28, CTLA4, EGR1, TLR2, etc.). Moreover, the analysis of pathways that were enriched in the target genes present in the clusters confirmed the essential role of these transcripts and their relative targeting miRNA in BD pathogenesis.

In conclusion, this work represents the first analysis performed on such a large number of miRNAs and integrated with the study of the profiles of gene expression in BD. The study allowed correlating the expression of miRNAs and the modulation of genes important for the pathogenesis of the disease. Using this approach, we have been able to identify the specific molecular pathways on which the regulation of these miRNAs may occur.

This study sheds light on some epigenetic aspects of BD identifying specific miRNAs, which may represent promising candidates for the identification of disease biomarkers and/or the design of novel therapeutic strategies in BD.

## Figures and Tables

**Table 1:** 

Patients utilized for gene array study	6 (100%)
Sex	
Male	4
Female	2
Clinical features	
Aphthous stomatitis	6 (100%)
Genital ulcers	2 (33%)
Erythema nodosum-like lesions	1 (17%)
Papulopustular lesion	5 (83%)
Uveitis	2 (33%)
Epididymitis	0
Neurological symptoms	0
Vasculitis	4 (67%)
Joint manifestation	3 (50%)
Gastrointestinal involvement	0
Association with HLA-B51	4 (67%)

**Table 2:** miRNAs modulated in BD patients versus healthy subjects.

ID	Probe set name	Transcript ID (array design)	Fold change	FDR *p* value	Accession
20518901	MIMAT0019041_st	hsa-miR-4505	9.46	0.0001	MIMAT0019041
20500781	MIMAT0004609_st	hsa-miR-149-3p	6.05	0.0003	MIMAT0004609
20500119	MIMAT0000065_st	hsa-let-7d-5p	−1.55	0.0066	MIMAT0000065
20500444	MIMAT0000256_st	hsa-miR-181a-5p	−1.76	0.0095	MIMAT0000256
20500778	MIMAT0000449_st	hsa-miR-146a-5p	−1.87	0.0032	MIMAT0000449
20501197	MIMAT0000703_st	hsa-miR-361-5p	−1.88	0.0013	MIMAT0000703
20503908	MIMAT0004780_st	hsa-miR-532-3p	−1.92	0.0032	MIMAT0004780
20502123	MIMAT0004748_st	hsa-miR-423-5p	−2	0.0052	MIMAT0004748
20501036	MIMAT0000617_st	hsa-miR-200c-3p	−2.12	0.0032	MIMAT0000617
20501182	MIMAT0000692_st	hsa-miR-30e-5p	−2.17	0.0081	MIMAT0000692
20500158	MIMAT0000085_st	hsa-miR-28-5p	−2.2	0.0016	MIMAT0000085
20500422	MIMAT0000244_st	hsa-miR-30c-5p	−2.49	0.0019	MIMAT0000244
20501276	MIMAT0000751_st	hsa-miR-330-3p	−2.53	0.0089	MIMAT0000751
20500797	MIMAT0000460_st	hsa-miR-194-5p	−2.6	0.0028	MIMAT0000460
20502124	MIMAT0001340_st	hsa-miR-423-3p	−2.61	0.0025	MIMAT0001340
20500159	MIMAT0004502_st	hsa-miR-28-3p	−2.66	0.0012	MIMAT0004502
20500718	MIMAT0000417_st	hsa-miR-15b-5p	−2.94	0.0005	MIMAT0000417
20500424	MIMAT0000245_st	hsa-miR-30d-5p	−2.99	0.0003	MIMAT0000245
20500795	MIMAT0004614_st	hsa-miR-193a-5p	−3.01	0.0015	MIMAT0004614
20500385	MIMAT0000222_st	hsa-miR-192-5p	−3.06	0.0019	MIMAT0000222
20500758	MIMAT0000438_st	hsa-miR-152-3p	−3.06	0.0046	MIMAT0000438
20500151	MIMAT0000081_st	hsa-miR-25-3p	−3.2	0.0003	MIMAT0000081
20503811	MIMAT0002821_st	hsa-miR-181d-5p	−3.3	0.0005	MIMAT0002821
20500123	MIMAT0000067_st	hsa-let-7f-5p	−3.34	0.0041	MIMAT0000067
20504274	MIMAT0003218_st	hsa-miR-92b-3p	−3.43	0.0018	MIMAT0003218
20500162	MIMAT0000087_st	hsa-miR-30a-5p	−3.74	0.0006	MIMAT0000087
20500488	MIMAT0000280_st	hsa-miR-223-3p	−4.32	0.0043	MIMAT0000280
20503887	MIMAT0002876_st	hsa-miR-505-3p	−4.58	0.0023	MIMAT0002876
20500733	MIMAT0000424_st	hsa-miR-128-3p	−5.07	0.0029	MIMAT0000424
20501291	MIMAT0000759_st	hsa-miR-148b-3p	−5.43	0.0084	MIMAT0000759
20501278	MIMAT0000752_st	hsa-miR-328-3p	−5.63	0.0029	MIMAT0000752
20500798	MIMAT0000461_st	hsa-miR-195-5p	−5.8	0.0012	MIMAT0000461
20500121	MIMAT0000066_st	hsa-let-7e-5p	−5.98	0.0052	MIMAT0000066
20500187	MIMAT0004514_st	hsa-miR-29b-1-5p	−6.81	0.0093	MIMAT0004514
20504378	MIMAT0003297_st	hsa-miR-628-3p	−6.83	0.0032	MIMAT0003297
20500170	MIMAT0004507_st	hsa-miR-92a-1-5p	−6.84	0.0005	MIMAT0004507
20500723	MIMAT0000419_st	hsa-miR-27b-3p	−7.22	0.0041	MIMAT0000419
20504553	MIMAT0004819_st	hsa-miR-671-3p	−8.16	0.0006	MIMAT0004819
20501287	MIMAT0000757_st	hsa-miR-151a-3p	−8.47	0.0084	MIMAT0000757
20503105	MIMAT0002177_st	hsa-miR-486-5p	−17.98	0.0066	MIMAT0002177
20500400	MIMAT0000232_st	hsa-miR-199a-3p	−21.75	0.0049	MIMAT0000232
20500458	MIMAT0004563_st	hsa-miR-199b-3p	−21.75	0.0049	MIMAT0004563
20500769	MIMAT0000445_st	hsa-miR-126-3p	−26.32	0.0023	MIMAT0000445
20504312	MIMAT0003249_st	hsa-miR-584-5p	−51.18	0.002	MIMAT0003249
20500399	MIMAT0000231_st	hsa-miR-199a-5p	−63.55	0.0023	MIMAT0000231
20500432	MIMAT0000250_st	hsa-miR-139-5p	−66.67	0.0027	MIMAT0000250
20500752	MIMAT0000435_st	hsa-miR-143-3p	−83.47	0.0046	MIMAT0000435

**Table 3:** List of all BD DEGs that were targeted by selected miRNAs modulated in BD and their respective targeting miRNAs.

DEGs	MicroRNA										
ACTR2	miR-181a-5p	miR-151-3p	miR-199a-3p	miR-199b-3p	miR-15b-5p	miR-195-5p					
AKIRIN2	miR-181a-5p	miR-27b-3p	miR-139-5p								
APLP2	miR-181a-5p	miR-139-5p	miR-199a-3p	miR-199b-3p							
ARF3	miR-181a-5p	miR-27b-3p									
BCL2L11	miR-181a-5p	miR-199a-3p	miR-199b-3p	miR-30e-5p	miR-30c-5p	miR-30d-5p	miR-181d-5p	miR-92b-3p	miR-30a-5p	miR-148b-3p	
BTG2	miR-181a-5p	miR-27b-3p	miR-199a-5p	let-7d-5p	miR-15b-5p	miR-25-3p	let-7f-5p	miR-92b-3p	miR-195-5p	let-7e-5p	miR-146a-5p
CALM1	miR-181a-5p	miR-139-5p	miR-143-3p	miR-151-3p	miR-199a-3p	miR-199b-3p	let-7d-5p	miR-181d-5p	let-7f-5p	miR-195-5p	let-7e-5p
CD4	miR-181a-5p										
CXCL2	miR-181a-5p										
DAZAP2	miR-181a-5p	miR-27b-3p	miR-139-5p	miR-151-3p	miR-29b-1-5p	miR-146a-5p					
DDX3X	miR-181a-5p	miR-27b-3p	miR-139-5p	miR-199a-5p	miR-671-3p	miR-195-5p	miR-146a-5p				
DNAJB1	miR-181a-5p	miR-27b-3p	miR-143-3p	miR-486-5p							
EGR1	miR-181a-5p	miR-143-3p	miR-199a-3p	miR-199b-3p	miR-146a-5p						
ERAP1	miR-181a-5p										
EREG	miR-181a-5p	miR-199a-3p	miR-199b-3p	miR-192-5p							
F5	miR-181a-5p										
HIPK1	miR-181a-5p	miR-139-5p	miR-143-3p	miR-199a-3p	miR-199a-5p	miR-199b-3p	miR-486-5p	miR-146a-5p			
HSP90B1	miR-181a-5p	miR-152-3p	miR-181d-5p	miR-223-3p	miR-148b-3p						
IL1A	miR-181a-5p	miR-181d-5p	miR-505-3p								
IL6ST	miR-181a-5p	miR-27b-3p	miR-143-3p	miR-152-3p	miR-223-3p	miR-505-3p	miR-148b-3p				
INPP4A	miR-181a-5p	miR-223-3p									
KMT2A	miR-181a-5p	miR-27b-3p	miR-199a-3p	miR-199b-3p	miR-148b-3p	miR-195-5p	miR-146a-5p				
LMAN1	miR-181a-5p	miR-152-3p	miR-148b-3p								
MARCKS	miR-181a-5p	miR-27b-3p	miR-143-3p	miR-199a-5p	miR-584-5p	miR-200c-3p	miR-128-3p				
MCL1	miR-181a-5p	miR-139-5p	miR-151-3p	miR-199a-3p	miR-199b-3p	miR-486-5p	miR-584-5p	miR-628-3p			
NKTR	miR-181a-5p	miR-27b-3p	miR-151-3p								
NR4A2	miR-181a-5p	miR-361-5p	miR-30e-5p	miR-30c-5p	miR-30d-5p	miR-30a-5p					
NR4A3	miR-181a-5p	miR-25-3p	miR-92b-3p								
PRRC2C	miR-181a-5p	miR-27b-3p	miR-486-5p	miR-195-5p							
SOCS3	miR-181a-5p	miR-30e-5p	miR-30c-5p	miR-30d-5p	miR-152-3p	miR-30a-5p	miR-148b-3p				
TNF	miR-181a-5p	miR-181d-5p									
YY1	miR-181a-5p	let-7f-5p	let-7e-5p								
ZFP36L2	miR-181a-5p	miR-27b-3p	miR-128-3p								
TNFAIP3	miR-27b-3p	let-7e-5p									
PLK2	miR-27b-3p	miR-126-3p	miR-128-3p	miR-146a-5p							
MAP4K5	miR-27b-3p	miR-128-3p									
HSPD1	miR-27b-3p	miR-143-3p	miR-628-3p	miR-505-3p							
IKZF1	miR-27b-3p	miR-199a-5p									
IL10	miR-27b-3p	let-7d-5p	let-7f-5p	let-7e-5p	miR-146a-5p						
CD28	miR-27b-3p	miR-143-3p	miR-15b-5p	miR-195-5p							
MLL	miR-27b-3p	miR-143-3p	miR-199a-3p	miR-199b-3p							
PIK3R1	miR-27b-3p	miR-126-3p	miR-199a-3p	miR-199b-3p	miR-486-5p	miR-15b-5p	miR-128-3p	miR-195-5p			
PTGER4	miR-27b-3p	miR-25-3p	miR-92b-3p	miR-146a-5p							
STAT1	miR-27b-3p	miR-584-5p	miR-128-3p	miR-146a-5p							
ICOS	miR-27b-3p	let-7d-5p	let-7f-5p	let-7e-5p							
KAT6A	miR-27b-3p	miR-139-5p	miR-143-3p								
VEGFA	miR-126-3p	miR-139-5p	miR-199a-3p	miR-199a-5p	miR-199b-3p	miR-486-5p	miR-361-5p	miR-15b-5p	miR-195-5p		
CXCR4	miR-139-5p	miR-143-3p									
JUN	miR-139-5p	miR-200c-3p									
AP2M1	miR-139-5p	miR-143-3p	miR-423-5p								
TCF3	miR-139-5p	miR-195-5p									
CREM	miR-139-5p	miR-199a-5p									
RBL2	miR-139-5p	miR-199a-3p	miR-628-3p								
TGIF1	miR-139-5p	miR-27b-3p	miR-199a-3p	miR-199b-3p	miR-25-3p	miR-92b-3p	miR-146a-5p				
THBD	miR-139-5p										
THBS1	miR-139-5p	miR-143-3p	miR-151-3p	miR-199a-3p	miR-199b-3p	miR-628-3p	let-7d-5p	miR-194-5p	let-7f-5p	let-7e-5p	
MMP8	miR-139-5p										
DDX6	miR-139-5p	miR-143-3p	miR-199a-5p	miR-152-3p	miR-148b-3p						
PGK1	miR-27b-3p	miR-143-3p	miR-146a-5p								
TSC22D3	miR-143-3p	miR-195-5p									
IL6R	miR-143-3p	let-7e-5p									
DUSP1	miR-143-3p	miR-199a-3p	let-7d-5p	miR-200c-3p	miR-152-3p	miR-25-3p	let-7f-5p	miR-148b-3p	let-7e-5p		
CD84	miR-143-3p										
CTLA4	miR-143-3p										
STAT2	miR-143-3p										
TLR2	miR-143-3p	miR-146a-5p									
PPP2R1A	miR-143-3p	miR-199a-5p	miR-15b-5p	miR-195-5p							
SIKE1	miR-143-3p	miR-199b-3p	miR-199a-3p	miR-146a-5p							
DUSP2	miR-143-3p										
FLOT1	miR-143-3p										
FOSL2	miR-143-3p	miR-30e-5p	miR-30c-5p	miR-30d-5p	miR-25-3p	miR-92b-3p	miR-30a-5p	miR-195-5p			
IKZF3	miR-151-3p	miR-146a-5p									
PTPN12	miR-151-3p	miR-486-5p	miR-194-5p								
NAMPT	miR-199a-3p	miR-199b-3p									
PDE4B	miR-199a-3p	miR-199b-3p	miR-152-3p	miR-148b-3p							
STK4	miR-199a-5p	miR-486-5p									
IKBKB	miR-199a-5p	miR-200c-3p	miR-15b-5p	miR-195-5p							
HSPA4	miR-199a-5p										
GNAI2	miR-29b-1-5p	miR-30e-5p	miR-30c-5p	miR-30d-5p	miR-30a-5p						
ZNF331	miR-486-5p	miR-146a-5p									
*PTX3*	miR-628-3p										
CCL3	let-7d-5p	let-7f-5p	let-7e-5p								
OLR1	let-7d-5p	let-7e-5p									
S100A8	let-7d-5p	let-7f-5p	let-7e-5p								
SOCS1	let-7d-5p	miR-30e-5p	miR-30c-5p	miR-30d-5p	let-7f-5p	miR-30a-5p	let-7e-5p				
*DGKA*	miR-200c-3p										
NAPA	miR-330-3p										
ARHGDIA	miR-15b-5p	miR-195-5p									
SNX27	miR-152-3p	miR-148b-3p									
NLRP3	miR-223-3p										
RHOB	miR-223-3p										
FLOT2	miR-148b-3p	miR-146a-5p									
RAB35	miR-148b-3p	miR-195-5p									
REL	let-7e-5p	miR-146a-5p									
KIR2DL4	miR-146a-5p										

**Table 4:** Pathways enriched in DEGs targeted in each cluster.

Panther pathways	*p* value
*CL1*	
Toll-like receptor signaling pathway	8.44*E* − 07
Apoptosis signaling pathway	2.34*E* − 05
Inflammation-mediated by chemokine and cytokine signaling pathway	3.09*E* − 05
Interleukin signaling pathway	1.68*E* − 04
PDGF signaling pathway	1.14*E* − 03
Oxidative stress response	1.46*E* − 03
B cell activation	1.80*E* − 03
T cell activation	3.49*E* − 03
JAK/STAT signaling pathway	3.81*E* − 03
Angiogenesis	1.79*E* − 02
*CL2*	
Wnt signaling pathway	1.20*E* − 02
Hypoxia response via HIF activation	1.87*E* − 02
Blood coagulation	2.03*E* − 02
Insulin/IGF pathway-protein kinase B signaling cascade	2.31*E* − 02
p53 pathway feedback loops 2	2.87*E* − 02
PI3 kinase pathway	3.15*E* − 02
VEGF signaling pathway	3.64*E* − 02
Endothelin signaling pathway	4.41*E* − 02
T cell activation	4.74*E* − 02
p53 pathway	4.90*E* − 02
*CL3*	
5HT1-type receptor-mediated signaling pathway	2.07*E* − 02
PI3 kinase pathway	2.63*E* − 02
Oxidative stress response	2.72*E* − 02
*CL4*	
Glycolysis	6.63*E* − 03
T cell activation	2.79*E* − 02
*CL5*	
PDGF signaling pathway	2.71*E* − 02
Angiogenesis	3.03*E* − 02
Integrin signaling pathway	3.04*E* − 02
*CL6*	
Cadherin signaling pathway	2.10*E* − 02

## Data Availability

The data used to support the findings of this study are available from the corresponding author upon request.

## Supplementary Materials

Supplementary 1 Supplementary Table 1: miRNAs significantly modulated in BD.

Supplementary 2 Supplementary Table 2: pathways enriched in genes targeted by modulated miRNAs in BD.

Supplementary 3 Supplementary Table 3: pathways enriched in differentially expressed genes in BD.

Supplementary 4 Supplementary Table 4: pathways enriched both in genes targeted by BD deregulated miRNAs and in BD differentially expressed genes.

Supplementary 5 Supplementary Table 5: differentially expressed genes in BD that were included in the six clusters.

Supplementary 6 Supplementary Table 6: differentially expressed genes in BD that were included in clusters that are targeted by modulated miRNAs in BD.

Supplementary 7 Supplementary Table 7: enriched pathways in target genes that are included in clusters.
